# Bone Marrow Assessment in Liver Cirrhosis Patients with Otherwise Unexplained Peripheral Blood Cytopenia

**DOI:** 10.3390/jcm12134373

**Published:** 2023-06-29

**Authors:** Sebastian E. Koschade, Laura M. Moser, Artur Sokolovskiy, Florian A. Michael, Hubert Serve, Christian H. Brandts, Fabian Finkelmeier, Stefan Zeuzem, Jonel Trebicka, Philip Ferstl, Olivier Ballo

**Affiliations:** 1 Department of Medicine, Hematology/Oncology, University Hospital Frankfurt, Goethe University, 60590 Frankfurt am Main, Germany; 2 Department for Children and Adolescents, University Hospital Frankfurt, Goethe University, 60590 Frankfurt am Main, Germany; 3 Department of Medicine, Gastroenterology, Hepatology and Endocrinology, University Hospital Frankfurt, Goethe University, 60590 Frankfurt am Main, Germany; 4 University Cancer Center Frankfurt (UCT), University Hospital Frankfurt, Goethe University, 60590 Frankfurt am Main, Germany; 5 Department of Internal Medicine B, University of Münster, 48149 Münster, Germany

**Keywords:** liver cirrhosis, bone marrow puncture, bone marrow aspiration, bone marrow biopsy, cytopenia

## Abstract

We performed a retrospective single-center analysis to investigate the diagnostic yield of bone marrow puncture in patients with liver cirrhosis and cytopenia. Liver cirrhosis patients receiving bone marrow aspiration or biopsy for the diagnostic work-up of otherwise unexplained peripheral blood cytopenia at our institution between 2004 and 2020 were enrolled in this study. We evaluated findings from cytologic, histologic and immunologic assessment and final diagnostic outcomes. A total of 118 patients with a median age of 55 years and a median Child–Pugh score of B (8 points) were enrolled. The main etiologies of liver cirrhosis were viral hepatitis (B and C) or chronic alcohol consumption. The majority of patients (60%) exhibited concurrent anemia, leukocytopenia and thrombocytopenia. Bone marrow assessment revealed normal, unspecific or reactive alterations in 117 out of 118 patients (99%). One patient was diagnosed with myelodysplastic syndrome. Our findings suggest that peripheral blood cytopenia in patients with liver cirrhosis is rarely associated with a primary bone marrow pathology.

## 1. Introduction

Peripheral blood cytopenias are frequently observed in patients with liver cirrhosis independent of etiology [1,2,3,4,5]. The frequency and severity of cytopenia and the number of affected lineages increase with the progression of liver cirrhosis [3,5,6]. Among compensated patients with liver cirrhosis, previous studies found abnormal hematologic indices in the majority of cases: thrombocytopenia in 64–77% of patients, leukopenia in 5–42% and anemia in 21–37% [1,3]. Thrombocytopenia develops first and may contribute to disease morbidity by increasing the risk of bleeding, followed by leukopenia and anemia [3].

The underlying reasons are often multifactorial and may partly be attributed to portal hypertension and hypersplenism with splenic sequestration and increased cell destruction, decreased serum erythropoietin levels, decreased hepatic thrombopoietin production with decreased hematopoiesis in the bone marrow, and blood loss from bleeding. Current recommendations include identifying and treating the underlying cause of cytopenia, such as managing bleeding from varices, correcting deficits in iron, folate acid or vitamin B12, and addressing portal hypertension [7,8].

However, peripheral blood cytopenias may also develop due to bone marrow pathologies arising from primary hematological diseases unrelated to liver cirrhosis, such as myelodysplastic syndrome (MDS), newly onset leukemia, or bone marrow involvement in other primarily extramedullary diseases. Although liver cirrhosis is a frequent cause of cytopenia, it may therefore be important to also consider alternative diagnoses which require bone marrow puncture for a definitive diagnostic work-up. Due to the frequent occurrence of hematologic abnormalities in liver cirrhosis patients, this constitutes a common clinical conundrum. Bone marrow punctures are invasive and painful diagnostic procedures that carry a risk of bleeding, infection and nerve damage [9,10,11,12]. In addition, liver cirrhosis patients are often at an increased risk of bleeding complications due to thrombocytopenia and reduced synthesis of clotting factors [7]. This underscores the importance of careful selection of patients undergoing bone marrow puncture. Current national [13,14] and international [15,16,17,18,19,20] guidelines and expert reviews [7,21] do not provide clear recommendations for bone marrow samples in these patients. There is a paucity of data regarding the utility of bone marrow punctures in the diagnostic work-up of liver cirrhosis patients presenting with cytopenia.

To address this question, a single-center retrospective study was conducted to evaluate all patients presenting with liver cirrhosis that underwent bone marrow puncture due to peripheral blood cytopenia. The primary outcome was the diagnostic yield of bone marrow puncture and secondary outcomes were group differences in patients with and without a newly diagnosed bone marrow disorder.

## 2. Materials and Methods

### 2.1. Study Population

Adult (age 18 years or older) patients with diagnosed liver cirrhosis as defined by clinical diagnosis and ultrasound/computed tomography (CT)/magnetic resonance (MR) imaging and/or histopathology or laboratory findings presenting at our institution between 2004–2020 who received a bone marrow puncture for the diagnostic work-up of otherwise unexplained peripheral blood cytopenia were retrospectively included. The screening period consisted of the period between diagnosis of liver cirrhosis and death, loss to follow-up, or liver transplantation. Patients with a known prior hematologic disease or patients who had received a liver transplant prior to bone marrow puncture were excluded, as well as patients with bone marrow puncture due to reasons other than cytopenia. No patients taking overtly bone marrow cytotoxic medications were included in the study. The study group was predefined. Patients were extracted from the digital patient registry of the University Clinic Frankfurt by ICD-10 code (diagnosis of liver cirrhosis) and German Procedure Classification (OPS; bone marrow puncture). Identified patients were manually annotated using their electronic health records. Demographic, clinical and laboratory data, including liver scores (Child–Pugh) from the time of bone marrow puncture, were recorded. The decision to gather bone marrow samples was made by the treating physician after consultation with the patient or his legal proxy. The local laboratory reference values for peripheral blood counts and hemoglobin were as follows: leukocytes 3.92/nl–9.81/nl (male) or 3.96/nl–10.41/nl (female); thrombocytes 146/nl–328/nl (male) or 176/nl–391/nl (female); erythrocytes 4.54/nl–5.77/nl (male) or 3.96/nl–5.16/nl (female); hemoglobin 13.5 g/dL–17.5 g/dL (male) or 11.6–15.5 g/dL (female). Patient data and consent to anonymized publication were provided after approval by the local ethics committee (ref. nr. UCT-17-2021) according to the 1964 Declaration of Helsinki and its later amendments.

### 2.2. Cytomorphology, Immunology, Histopathology

Bone marrow biopsies and aspirates were obtained and analyzed according to the hospital’s standard procedures. Bone marrow aspirates and biopsies were obtained from the posterior superior iliac spine using a dedicated, single-use bone marrow aspiration or core biopsy needle. Smears were prepared immediately after aspiration and were subsequently air-dried and stained with May-Grünwald-Giemsa staining in accordance with the ICSH guidelines [22]. Bone marrow core needle biopsies were immediately placed into a formaldehyde solution. Only bone marrow aspirates containing particles were further considered for cytologic assessment. Slides were visualized with a Zeiss Axioskop 2 plus light microscope using a 63× Zeiss oil immersion objective. The in-house cytomorphological assessment was carried out independently by two experienced investigators, a senior attending physician of the Department of Hematology/Oncology and an experienced technician. Bone marrow biopsies were decalcified, embedded in paraffin, sectioned and stained (Alcian blue and PAS staining, Berlin blue staining, silver staining, lysozyme and MPO staining, antibody staining with Anti-CD61, Anti-CD138, Anti-CD3, Anti-CD20, Anti-CD79a, Anti-CD34, Anti-CD117, Anti-TdT; additional markers as needed) and routine histopathological assessment was performed by a senior pathologist in-house. Immunologic assessment of bone marrow aspirates using multicolor flow cytometry (FSC, SSC, typically Anti-CD45, Anti-CD34, Anti-CD-117, Anti-HLA-DR, Anti-CD-13, Anti-CD-7, Anti-CD56, Anti-CD19, Anti-CD20, Anti-CD38, Anti-SmIgKappa, Anti-SmIgLambda; further antibodies as required) was likewise performed in-house. Cytogenetic and molecular genetic analysis was conducted by Münchner Leukämie Labor (MLL) GmbH, Munich, Germany.

### 2.3. Statistical Analysis

Results are shown as the median and interquartile range for continuous variables and as absolute numbers and percentages for categorical variables. Linear regression analysis of lineage counts on Child–Pugh score (without categorization into groups) was performed using robust regression (lmrob) implemented in R’s robustbase 0.95–1 package using default settings. R 4.2.2 and ggplot2 3.4.0 were used for statistical analyses, data reporting and plotting. A *p* value < 0.05 was considered statistically significant.

## 3. Results

### 3.1. Baseline Characteristics

**Figure 1:**
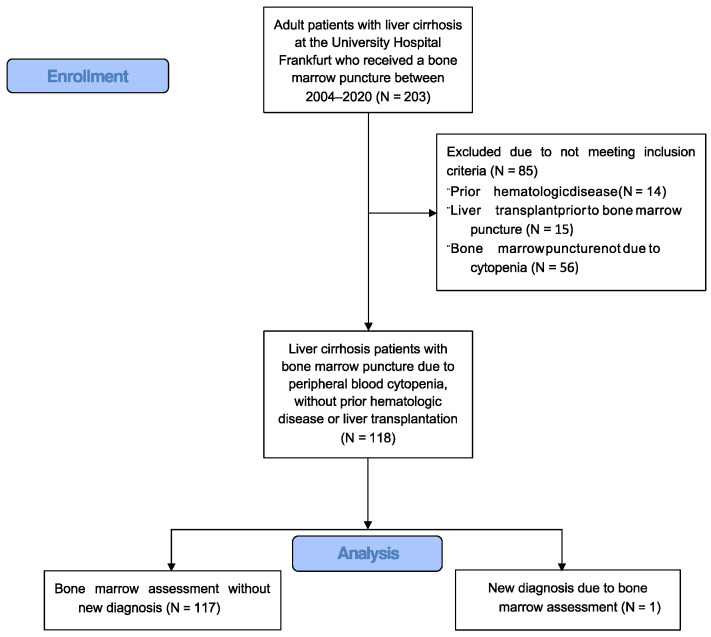
Study Flow Diagram.

The study flow diagram is shown in Figure 1. A total of 118 patients with liver cirrhosis who underwent bone marrow puncture for the diagnostic work-up of peripheral blood cytopenia between 2004 and 2020 were identified. Table 1 shows the baseline and laboratory characteristics, etiology of liver cirrhosis, concomitant diseases, Child–Pugh score and overall survival. Hepatitis C virus (HCV) was the most frequent etiology of liver disease (*n* = 43, 36%), followed by chronic alcohol consumption (*n* = 32, 27%). Diabetes mellitus and chronic kidney disease were concomitant diseases in 26% (*n* = 31) and 20% (*n* = 24), respectively. The median Child–Pugh score was B (8 points). Ascites was present in 56% of cases (*n* = 66) and hepatic encephalopathy in 16% (*n* = 19). Among patients with viral etiology of liver cirrhosis, 53% (*n* = 32) had received prior antiviral therapy, which was discontinued prior to bone marrow assessment in 63% (*n* = 20), with a median time between the end of antiviral treatment to bone marrow puncture of 20 months (inter-quartile range (IQR) 3–67 months). The median overall survival of liver cirrhosis patients after bone marrow puncture was 30 months (IQR 12–82 months).

### 3.2. Peripheral Blood Cytopenias

Peripheral blood counts of patients with liver cirrhosis prior to bone marrow puncture are shown in Table 1. Details on peripheral blood lineages affected by cytopenia are shown in Table 2. All 118 patients had peripheral blood cytopenia per the study group’s inclusion criteria. Most patients had concomitant cytopenia in the erythroid, leukocyte and thrombocyte lineages (pancytopenia, 60%). The lineages most frequently affected by cytopenia were the erythroid (93%) and thrombocyte (92%) lineages. No patient had received specific drug treatments for cytopenia.

**Figure 2:**
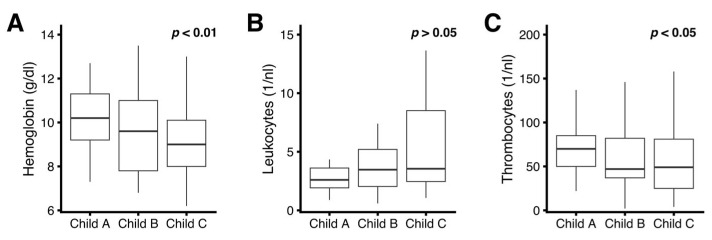
Correlation of liver disease severity (Child–Pugh score) with cytopenia. (**A**) Hemoglobin; (**B**) Total peripheral blood leukocyte count; (**C**) platelet count. Data are summarized for individual Child–Pugh groups by boxplots (horizontal bar inside boxplot indicates median, box extends from the 25th to the 75th percentile, whiskers extend to the highest/lowest value within 1.5× inter-quartile range of the data. *p* values for overall dependency of individual lineage count on liver disease severity were obtained by robust linear regression on the non-categorized Child Pugh score.

Peripheral blood cytopenia correlated with the severity of liver disease for hemoglobin levels and platelet counts: on average, a higher Child–Pugh score was associated with lower hemoglobin levels and lower platelet counts (Figure 2A,C). Total leukocyte counts were abnormally decreased in liver cirrhosis patients regardless of the Child–Pugh Score; however, no statistically significant correlation with Child–Pugh score was found (Figure 2B).

### 3.3. Bone Marrow Evaluations

All patients underwent bone marrow puncture for further diagnostic evaluation of peripheral blood cytopenia. The results of the bone marrow evaluations are displayed in Table 3. Both cytologic and histopathologic assessments were performed in the majority of cases (91%). Immunologic evaluation by flow cytometry was performed in 54% of patients. Cytogenetic or molecular genetic assessments were performed in a small minority of patients (4% and 3%, respectively). The majority of patients (76%) did not exhibit cytologic signs of dysplasia. Cytologic assessment yielded either a normal result or noted unspecific alterations in the majority of patients (40% and 26%, respectively). In 16% of patients, hyper- or hypoplastic myelopoiesis was noted. A total of 5% of patients were noted to exhibit bone marrow myelodysplasia without meeting the diagnostic criteria for MDS [23], and one patient (1%) was diagnosed with MDS. Histopathologic evaluation similarly noted mostly normal or unspecific/reactive changes (17% and 63% of patients, respectively) without any formal disease diagnosis. Myelodysplasia (without meeting diagnostic MDS criteria) and maturation defects were noted in 5% of patients. Further immunologic evaluation by flow cytometry did not add diagnostic insight. Lymphoma infiltration suspected by cytology (2% of patients) or flow cytometry (3% of patients) was not confirmed upon histopathologic assessment.

### 3.4. Clinical Impact of Bone Marrow Evaluations

Evaluation by bone marrow puncture did not yield a new diagnosis in 117 out of 118 (99.2%) patients with liver cirrhosis and peripheral blood cytopenia. One patient was diagnosed with MDS but had not further progressed or developed transfusion dependency or indication for treatment until data cut-off at the last follow-up.

Since only one patient with a new diagnosis made by bone marrow puncture was identified in our study, no group comparisons between patients with and without a newly diagnosed bone marrow disorder were attempted.

## 4. Discussion

The aim of this retrospective study was to evaluate the diagnostic utility of bone marrow punctures in patients with liver cirrhosis. Our study found that the diagnostic yield of bone marrow punctures was low, and that bone marrow evaluation did not result in any management changes for the majority of patients. This suggests that peripheral blood cytopenias in patients with liver cirrhosis are, in most cases, not indicative of an underlying bone marrow disease and further suggests that the routine use of bone marrow punctures in patients with liver cirrhosis and cytopenia may not be necessary.

Since we included only patients with peripheral blood cytopenia, the frequency and severity of peripheral blood cytopenias in our cohort were higher than those reported in studies that included all patients with advanced liver cirrhosis [24]. In agreement with our findings, the limited number of available reports on bone marrow assessment in patients with liver cirrhosis likewise identified non-specific or reactive changes that did not meet diagnostic criteria for primary bone marrow pathologies in most cases [25,26,27,28]. To our knowledge, our study is the first to report results of a comprehensive, combined cytologic, histologic and immunologic bone marrow assessment in patients with liver cirrhosis and peripheral blood cytopenia.

Our results are contingent upon the medical considerations that led to the bone marrow punctures in our study group. Generally, bone marrow puncture was part of the diagnostic work-up in newly presenting patients with a first diagnosis of severe liver cirrhosis and cytopenia when the order of onset (liver cirrhosis first vs. pre-existing cytopenia) remained unclear. Bone marrow puncture was also performed when the degree of peripheral blood cytopenia was perceived to be disproportional to the severity of liver cirrhosis. We did not identify patients with a prior known diagnosis of liver cirrhosis in our study cohort who underwent bone marrow puncture due to newly onset, acute cytopenia.

Our retrospective single-center study has several limitations. The study group size is moderate. Cytomorphologic, histopathologic and immunologic assessments rely on the expertise of individual investigators, which may limit the generalizability of our results. Patients included in this study underwent bone marrow puncture at the discretion of the treating physician, and our study thus represents a real-world description of this diagnostic procedure in such a patient cohort. However, this may have biased our study results. It is possible that a more systematized bone marrow assessment of liver cirrhosis patients would have yielded different results. Further, we have not systematically assessed the functional consequences of cytopenia, such as symptoms of anemia, frequency of hemorrhage or infection. Likewise, we have not addressed possible therapeutic interventions such as the use of thrombopoietin [29] receptor agonists [30,31] originally developed for immune thrombocytopenia [32,33], which may have good efficacy in cytopenic patients with a normal bone marrow evaluation. For a better understanding of the pathophysiology of cytopenia in liver disease, it might be informative to perform myeloid panel NGS sequencing as well as cytogenetics on a larger percentage of patients. However, this has not been conducted here.

In addition, it is important to highlight that patients who underwent bone marrow puncture due to increased peripheral blood counts or gross abnormalities in peripheral blood smears (such as circulating myeloid or lymphatic blasts) were not included in our study. Our results, therefore, do not apply to this patient group. Patients with a known prior hematologic disease were also not included. Therefore, our study does not reflect upon the frequency of concomitant hematologic disease in all patients with liver cirrhosis. Finally, the etiology of liver cirrhosis in our cohort had been established in almost all cases. Therefore, patients with liver cirrhosis of unknown etiology who may be at risk of rare diseases presenting with bone marrow failure and liver cirrhosis may not have been represented in the present study [34,35].

In summary, our study suggests that isolated peripheral blood cytopenia does not add substantial probability to the existence of a primary bone marrow pathology in patients with liver cirrhosis. Further studies are required to confirm this finding and to better define the subset of patients who may benefit from bone marrow punctures, such as patients with newly onset cytopenia, the cryptic origin of both liver cirrhosis and cytopenia, and patients with gross morphologic abnormalities in peripheral blood smears.

## Figures and Tables

**Table 1:** Baseline characteristics.

		N (%)
All patients		118 (100%)
Gender	Female	46 (39%)
	Male	72 (61%)
Age, years		55 (48–64)
Etiology	ASH	32 (27%)
	NASH	6 (5%)
	HBV	17 (14%)
	HCV	43 (36%)
	PBC	3 (3%)
	AIH	8 (7%)
	Toxic, non-alcohol	2 (2%)
	Cirrhose cardiaque	1 (1%)
	Kryptic	1 (1%)
	Other	5 (4%)
Concomitant diseases	Diabetes mellitus	31 (26%)
	Chronic kidney disease	24 (20%)
	Heart failure	9 (8%)
	COPD	8 (7%)
Child–Pugh Score		8 (7–10)
	Child A	25 (21%)
	Child B	51 (43%)
	Child C	33 (28%)
	Missing data	9 (8%)
Ascites	No	52 (44%)
	Moderate	14 (12%)
	Massive	52 (44%)
Encephalopathy	No	99 (84%)
	Grade I–II	18 (15%)
	Grade III–IV	1 (1%)
Spleen length, cm		13.7 (16.0–18.2)
Laboratory values	Albumin, g/dL	3.1 (2.7–3.5)
	Bilirubin, mg/dL	1.7 (0.9–3.9)
	INR	1.4 (1.2–1.7)
	Creatinine, g/dL	1.0 (0.7–1.6)
	LDH, U/L	234 (185–299)
	Hemoglobin, g/dL	9.6 (8.3–11.0)
	Erythrocytes, /pL	3.3 (2.8–3.8)
	Leukocytes, /nl	3.3 (2.2–4.9)
	Thrombocytes, /nl	58 (37–82)
Overall survival, months		30 (12–82)

Count data is shown for categorical variables. Continuous data are summarized by median value and inter-quartile range (IQR). ASH, alcoholic steatohepatitis; NASH, non-alcoholic steatohepatitis; HBV, hepatitis B virus; HCV, hepatitis C virus; PBC, primary biliary cholangitis; AIH, autoimmune hepatitis; COPD, chronic obstructive pulmonary disease; INR, international normalized ratio; LDH, lactate dehydrogenase.

**Table 2:** Details on peripheral blood cytopenias.

No. of Lineages Affected	No. of Patients (N = 118)	Affected Lineage(s)		
		Erythroid (N = 110, 93%)	Leukocytes (N = 74, 63%)	Thrombocytes (N = 108, 92%)
1/3	11 (9%)	6 (5%) Hb (g/dL) 9.6 [7.8–13.4]	0 (0%)	5 (4%) PLT/nl 74 (50–114)
2/3	34 (29%)	33 (28%) Hb (g/dL) 9.0 [6.9–13.5]	3 (3%) TLC/nl 3.11 [1.48–3.53]	32 (27%) PLT/nl 54 (2–145)
3/3	71 (60%)	71 (60%) Hb (g/dL) 9.6 [6.2–13.1]	71 (60%) TLC/nl 2.46 [0.59–3.77]	71 (60%) PLT/nl 50 (11–137)

Patient numbers with affected lineages and median values for hemoglobin (Hb), total leukocyte count (TLC) and platelet count (PLT) with data ranges are shown. For two patients with peripheral blood cytopenia, precise blood counts were not retrievable. No., number.

**Table 3:** Bone marrow evaluations.

			N (%)
BMA/BMP due to cytopenia			118 (100%)
Type of assessment	Cytology		107 (91%)
	Histopathology		107 (91%)
	Immunology		64 (54%)
	Cytogenetics		5 (4%)
	Molecular genetics		3 (3%)
Cytologic signs of dysplasia	yes		17 (14%)
		Erythropoiesis	13 (11%)
		Megakaryopoiesis	8 (7%)
		Granulopoiesis	4 (3%)
	no		90 (76%)
Assessment	Cytology	Normal	47 (40%)
		Unspecific/reactive	31 (26%)
		Hyperplastic myelopoiesis	12 (10%)
		Hypoplastic myelopoiesis	7 (6%)
		Myelodysplasia without MDS	6 (5%)
		Suspected lymphoma	2 (2%)
		MDS	1 (1%)
	Histopathology	Normal	20 (17%)
		Unspecific/reactive	74 (63%)
		Hyperplastic myelopoiesis	5 (4%)
		Hypoplastic myelopoiesis	2 (2%)
		Myelodysplasia without MDS	3 (3%)
		Maturation defect	2 (2%)
	Immunology	Normal	56 (47%)
		Unspecific/reactive	4 (3%)
		Suspected lymphoma	3 (3%)
	Cytogenetics	Normal	5 (4%)
	Molecular genetics	Normal	3 (3%)
Final diagnosis		No new diagnosis	117 (99%)
		MDS	1 (1%)

Count data is shown. BMA, bone marrow aspiration; BMB, bone marrow biopsy; MDS, myelodysplastic syndrome.

## Data Availability

The data presented in this study are available on reasonable request from the corresponding author. The data are not publicly available due to privacy restrictions.
